# Associations between frequency of physical exercise and global cognition among community‐dwelling Latino older adults from the Boston Latino Aging Study

**DOI:** 10.1002/alz70858_104069

**Published:** 2025-12-26

**Authors:** Randy Medrano, Lusiana Martinez, Nadeshka J Ramirez‐Perez, Alexandre Closs Fanfa Ribas, Diana Munera, Jairo E. Martinez, Averi Giudicessi, Jorge Alcina, Isabel Solis, Clara Vila‐Castelar, Marta Gonzalez Catalan, Liliana A Ramirez Gomez, Daniel G Saldana, Yakeel T. Quiroz

**Affiliations:** ^1^ Massachusetts General Hospital, Harvard Medical School, Boston, MA, USA; ^2^ Department of Psychological & Brain Sciences, Boston University, Boston, MA, USA; ^3^ Harvard Medical School, Boston, MA, USA; ^4^ Boston University, Boston, MA, USA; ^5^ Massachusetts General Hospital, Harvard Medical School, Charlestown, MA, USA

## Abstract

**Background:**

Latinos are 1.5 times more likely than non‐Latino Whites to develop Alzheimer's Disease and Related Dementias (ADRD). This increased risk is partly attributable to a higher prevalence of risk factors, though the exact connection remains unclear. Physical activity (PA) has been recognized as a protective factor in preserving cognitive health and mitigating ADRD risk. While prior research has explored this relationship among non‐Latino Whites, there is limited evidence on the protective role of PA in older Latino adults. This study investigates the associations between PA frequency and global cognition in community‐dwelling older Latinos.

**Method:**

A total of 95 Spanish‐speaking participants (M_Age_ 65.06 ± 6.65, M_education_ 12.16 ± 4.88 years, 70.5% female, and 80 cognitively unimpaired) from the Boston Latino Aging Study (BLAST) were included. PA frequency was assessed by the Community Healthy Activities Model Program for Seniors (CHAMPS), a self‐reported questionnaire, which measured total minutes per week of various forms of exercise. Global cognition was evaluated with the Mini Mental State Examination (MMSE). Spearman rank correlations examined the relationship between physical activity and global cognition. Non‐parametric partial correlations were performed to adjust for age, sex, education and cardiac risk ratio‐ calculated as total cholesterol divided by HDL cholesterol, a marker of atherosclerosis risk.

**Result:**

On average, pand had a mean MMSE score of 26.68 ± 2.98. A higher frequency of physical activity was significantly associated with better performance on the MMSE (*r*_s_ = .25, *p* = .02), even after adjusting for covariates.

**Conclusion:**

A higher frequency of weekly physical activity was linked to better global cognition among older Latino adults, highlighting the protective role of regular physical activity in enhancing cognitive performance and reducing cardiovascular risk within this population. Our findings support the inclusion of physical activity as an integral part of Alzheimer's prevention strategies for Latinos. Future longitudinal studies with larger, more diverse samples and objective PA measures is needed to further elucidate the relationship between physical activity and cognition in Latinos and other ethno‐racially diverse groups.

